# Evaluation of the feeding safety of Moringa (*Moringa oleifera* L.) in the Sprague Dawley rat

**DOI:** 10.1038/s41598-024-51442-8

**Published:** 2024-05-09

**Authors:** Yu-Wen Zhang, Fu-Jun Wang, Ming Cai, Yan-Pei Liu, Jian-Yong Liu, Bi-Zhi Huang

**Affiliations:** 1 Yunnan Forestry Technological College, Kunming, 650224 Yunnan China; 2 Academe of Grassland and Animal Science, Kunming, 650212 Yunnan China

**Keywords:** Mouse, Energy supply and demand

## Abstract

This study aimed to evaluate the safety of Moringa by comparing the effects of different gavage doses of Moringa. The general behavior, body weight, food intake, blood indexes, serum biochemical indexes, and histopathology of rats were used to determine the safety threshold and to provide a reference for the further development and use of Moringa as animal feed. 40 Sprague Dawley rats were selected and given transoral gavage for 28 consecutive days. The T1, T2 and T3 groups were observed for general behavior, body weight, and food intake. Blood and serum biochemical indices were quantified, and histopathology was performed to evaluate the effect and safety of Moringa. The results of the toxicological test showed that (1) Only T1 groups experienced diarrhea. (2) The body weight and food intake of rats in each group were normal compared with the control group. (3) The hematological and serum biochemical indices of rats in the T1 group were significantly different from those of CK but were in the normal range; (4) The results of microscopic examination of the heart, liver, spleen, lung, and kidney of rats in each group were normal, but inflammation occurred in stomach and jejunum of rats in the T1 group, but not in the ileum. The gastrointestinal tract of rats in the T2 and T3 groups were normal. (5) No abnormal death occurred in any of the treatment groups.The results of this study revealed that gavage of Moringa homogenate at a dose of 6 g/kg BW can cause diarrhea in rats. Although there is no pathological effect on weight, food intake, blood and serum biochemical indicators in rats, there are pathological textures in the gastrointestinal tissue caused by diarrhea. Therefore, the safety threshold of Moringa homogenate should be ≤ 3 g/kg BW.

## Introduction

Chinese grasslands are an important component of Eurasian grasslands with 70 million years of history, covering an area of 392.8 million ha, accounting for 40.9% of China’s land area (State Forestry and Grassland Administration of China). China is rich in forage resources, with 6704 species of grass forage plants alone^1^. However, many resources are on the verge of extinction due to a lack of protection, coupled with species invasion, environmental pollution, and urbanization, which have exacerbated the decline in forage species^2^. With the increased demand for protein-based foods in the national dietary structure, the demand for forage feeds, meat, eggs, and milk in China has increased. The shortage of forage has become a major difficulty in food supply and security. Therefore, the development and utilization of high-quality forage feed have an important role in the development of China’s livestock industry.

Moringa (*Moringa oleifera* L.), commonly known as horseradish, is a perennial tropical deciduous tree known for its pungent roots and is native to northern India. It is widely grown in tropical and subtropical regions^3^ and has frost- and drought-resistant properties^4^. Moringa was first introduced to China with the continuous eastward migration of Indian Buddhism in the thirteenth century^5^. Moringa has a high protein content and a wide variety of amino acids^6–8^. It is also rich in trace elements such as potassium, calcium, and iron, which are beneficial for maintaining normal physiological functions in humans and animals. Currently, the use of Moringa is increasing in livestock feed, Moringa fed to poultry reduces egg serum cholesterol and triglyceride levels^9^, improves lactation in rabbits^10^,improves shrimp growth rate, protein utilization and antioxidant properties^11^, and is sufficient to increase milk and goat milk production and quality^12,13^, but some studies have shown that inappropriate ratios of Moringa in the diets of livestock animals can negatively affect growth performance, weight gain, intake, and health of animals, and in severe cases, can cause diarrhea. However, there is a paucity of research in this area, so it is important to assess the safety of moringa as a feed.

To establish the Moringa as a food source, its safety must first be determined. In this study, we gave fresh and silage homogenates of Moringa to Sprague Dawley (SD) rats by oral gavage for 28 consecutive days, once daily. The effects of Moringa on the general behavior, body weight and food intake, hematology, serum biochemistry, and histopathology of rats were observed, and the maximum no observed adverse effect level was determined.

## Materials and methods

### Sampling and pretreatment

Moringa was harvested from the Institute of Thermal Zone Ecological Agriculture, Yunnan Academy of Agricultural Sciences (Yunnan, China), in June 2021. The tested plants in this study are non endangered species, and the sample collection method is based on the principle of not damaging the ecological environment.

Homogenate preparation: 200 g of Moringa stems and leaves were weighed, added to 500 ml carboxymethylcellulose (CMC), and blended in a blender for 10 min. The suspension was used to make a high dose (400 mg/ml; T1), a medium dose (200 mg/ml; T2), and a low dose (100 mg/ml; T3) gavage sample. Samples were used for a maximum of 4 days and were mixed evenly with ultrasonic homogenization before intragastric administration via oral gavage. CMC (0.5% aqueous solution) was administered to control animals (CK) by oral gavage.

### Experimental animals

A total of 60 SD rats were purchased from Spelford (Beijing) Biotechnology Co., Ltd (Laboratory Animal Production License No. SCXK (Beijing) 2019-0010).SD rats were half male and half female, with a body weight of 239.3 ± 4.7.

### Feeding environment and management

Animals were raised in a general animal laboratory environment with a temperature of 20–26 °C (daily temperature difference ≤ 4 °C) and a humidity of 40–70%. Animals were kept in a transparent rat cage 47 × 31.5 × 20 cm (length × width × height) and were fed SPF-grade rat growth breeding feed (Beijing KeoXieLi Feed Co, Ltd). The drinking water was homemade drinking water, which was autoclaved for the animals to drink freely.

### Experimental treatments and toxicity assessment

Sixty SD rats were acclimatized for 7 days before gavage administration, during which clinical observation and recording were performed once daily. At the end of the acclimatization period, 40 rats were selected to enter the test after eliminating animals with abnormal conditions and too large or too small body weights. The animals were randomly divided into four groups of ten animals each (Table 1).

**Table 1:** Grouping of toxicological tests on rats.

	Groups	Test material	Density (g/ml)	Volume (ml/kg)	Dosage (g/kg)	Number of animals (n)
T1	Fresh Moringa homogenate high-dose group	Fresh Moringa + 0.5% CMC homogenate suspension	0.4	15	6	10
T2	Fresh Moringa homogenate medium-dose group		0.2	15	3	10
T3	Fresh Moringa homogenate low-dose group		0.1	15	1.5	10
CK	Solvent control group	0.5% CMC aqueous solution	0	15	0	10

Rats were gavaged once a day, weighed twice a week, and fed rat growth breeding feed once a week for 28 days. Clinical observations were recorded once a day, including diet, behavioral activity, mental condition, fur condition, glandular secretion, respiration, fecal properties, local reactions, death, and symptoms of toxic reactions, including their onset, severity, duration, reversibility, and recovery time. At the end of the test period, blood and serum samples were collected from female rats for clinical tests.

At the end of the gavage period, the rats were killed and the organs of the rats in the high-dose group were observed first. If obvious histopathological changes or abnormalities were found in the high dose group, the relevant tissues or organs were examined in the medium and low dose groups. The heart, liver, spleen, lung, kidney, stomach and intestinal systems of the rats with lesions were removed and fixed in 10% neutral formalin fixative solution for more than 1 week, then sampling, sectioning, HE staining, full slide scanning and reading.

### Statistical analysis

One-way ANOVA was performed using SPSS 17.0 statistical software for Windows. Multiple comparisons were performed using a Duncan multiple-range test when differences were significant. Results are expressed as mean ± standard deviation using *p* < 0.05 as the level of statistical significance.

### Ethics approval and consent to participate

The animal management and experimental procedures of this study were conducted in accordance with the ARRIVE guidelines (PLoS Bio 8 (6), e10004122010) and approved by the Life Science Ethics Review Committee of Yunnan Agricultural University, with an ethics code of 202,209,005. In this study, according to the “Guidelines for euthanasia of experimental animals” (GB/T 39,760-2021), carbon dioxide was used to kill rats and every effort was made to minimize their pain. All methods were carried out in accordance with relevant guideline and regulations.

## Results

### Mortality and general behavior

The mental condition of the rats in the fresh Moringa homogenate high-dose group was average during the gavage period, and the coat was rough (Table 2). However, the mobility and response were normal, and the rats showed slight diarrhea from day 1 to day 8 of gavage and severe diarrhea from day 9 to day 28 of gavage. The rats in the fresh Moringa homogenate medium and low-dose groups had good mental status, normal coat, mobility and reaction, and no diarrhea during the whole gavage period.

**Table 2:** General behavioral and diarrhea status of rats during the experimental period.

Groups	Treatment	General behavior	Fecal	
T1	Fresh Moringa Homogenate High-dose group	The coat is rough, and the mental condition is average Normal movement and reaction	1–8 d, slightly pale green stools, shaped but slightly watery, slight diarrhea;	9–28 d, slightly light green, shapeless, thin stools, diarrhea
T2	Fresh Moringa Homogenate Medium-dose group	Good mental and coat condition Normal movement and reaction	1–28 d, feces brown, shaped, no diarrhea	-
T3	Fresh Moringa Homogenate Low-dose group	Good mental and coat condition Normal movement and reaction	1–28 d, feces brown, shaped, no diarrhea	-

### Body weight

The body weight of rats in all groups increased gradually with longer gavage time, and no abnormalities were observed (Table 3). The initial weight, final weight, and mean daily weight gain of the rats were not significantly different compared to the control group (*P* > 0.05), indicating no adverse effect of different doses of fresh Moringa on the weight gain.

**Table 3:** Effect of Moringa on body weight of rats during the experimental period.

Items	Treatments			
	CK	T1	T2	T3
Initial Body Weight (g)	239.83 ± 6.5	239.42 ± 4.68	240.23 ± 5.7	239.52 ± 5.76
Final Body Weight (g)	347.81 ± 23.59	341.8 ± 21.32	356.41 ± 23.85	355.81 ± 22.08
Average Daily Gain (g·d^−1^)	3.86 ± 0.63	3.66 ± 0.61	4.15 ± 0.66	4.15 ± 0.61

The results were expressed as mean ± standard deviation and marked as significant level.

### Food intake

Food intake was calculated as the difference between the given amount of feed provided at the beginning of the test period and the amount of feed remaining at the end of one 4-day cycle. One value was recorded separately for each group per feed cycle. No significant difference (*P* > 0.05) was observed in the amount of food ingested by all four groups during the gavage period (Table 4).

**Table 4:** Effect of Moringa on food intake of rats during the experimental period.

Time(d)	Treatments		
	CK	T1	T2
0	–	–	–
4	105.6 ± 6.16	104.95 ± 6.55	106.1 ± 6.35
8	76.12 ± 4.34	72.75 ± 3.97	73.1 ± 4.15
12	101.37 ± 5.47	97.37 ± 5.76	98.52 ± 5.51
16	171.37 ± 11.21	163.05 ± 7.16	159.54 ± 7.14
20	73.45 ± 4.01	68.48 ± 4.01	70.42 ± 3.8
24	97.67 ± 5.06	95.83 ± 5.44	97.88 ± 5.04
28	73.17 ± 4.54	64.62 ± 3.94	67.23 ± 4.54
Average daily intake (g d^−1^)	22.45 ± 1.45	21.33 ± 1.31	21.53 ± 1.3

The results were expressed as mean ± standard deviation and marked as significant level.

### Hematology and serum biochemistry

All hematological indices of all groups during the gavage period were within the normal physiological range (Table 5). The levels of platelet (PLT), mean platelet volume (MPV) and mean corpuscular hemoglobin concentration (MCHC) in the blood of T1 rats were significantly lower than those of CK (*P* < 0.05). The blood indices of T2 and T3 rats were not significantly different from those of CK.

**Table 5:** Effect of Moringa on blood parameters of rats during the experimental period.

Hematological indices	Treatments			
	CK	T1	T2	T3
WBC (10^9^/l)	4.51 ± 0.35a	5.05 ± 0.47a	5.66 ± 0.74a	4.55 ± 0.61a
PLT (10^9^/l)	809.1 ± 13.88a	721.7 ± 22.85b	754.8 ± 25.41ab	746.5 ± 25.78ab
NEU (10^9^/l)	0.95 ± 0.12a	0.74 ± 0.04a	0.89 ± 0.13a	1.01 ± 0.14a
LYM (10^9^/l)	3.27 ± 0.28a	4.03 ± 0.44a	4.44 ± 0.71a	3.19 ± 0.48a
MON (10^9^/l)	0.2 ± 0.02a	0.19 ± 0.04a	0.26 ± 0.05a	0.28 ± 0.07a
EOS (10^9^/l)	0.08 ± 0.01a	0.08 ± 0.01a	0.07 ± 0.01a	0.07 ± 0.01a
RBC (10^12^/l)	8.11 ± 0.21a	8.36 ± 0.26a	8.06 ± 0.25a	8.09 ± 0.29a
HGB (g/l)	150.1 ± 3.09a	155.9 ± 3.8a	152.1 ± 3.66a	150.8 ± 3.53a
HCT (%)	45.15 ± 1.06a	47.95 ± 1.31a	46.03 ± 1.26a	45.64 ± 1.14a
MCV (fl)	55.71 ± 0.61a	57.52 ± 0.86a	57.18 ± 0.75a	56.65 ± 0.95a
MCH (pg)	18.53 ± 0.22a	18.74 ± 0.27a	18.93 ± 0.27a	18.72 ± 0.3a
MCHC (g/l)	332.5 ± 1.38a	325.9 ± 1.81b	331 ± 1.78a	330.3 ± 1.61a
MPV (fl)	15.08 ± 0.02a	11.85 ± 1.34bc	15.08 ± 0.04a	15.17 ± 0.03a

The results are expressed as mean ± standard deviation. Different lowercase letters in the same parameter show significant differences (*P* < 0.05).

*WBC* white blood cell count, *NEU* neutrophil count, *LYM* lymphocytes, *MON* monocytes, *EOS* eosinophil count, *RBC* red blood cell count, *HGB* hemoglobin, *HCT* hematocrit, *MCV* mean corpuscular volume, *MCH* mean corpuscular hemoglobin, *MCHC* mean corpuscular hemoglobin concentration, *MPV* mean platelet volume, *PLT* platelet.

All the serum biochemical indices of all groups during the gavage period were within the normal physiological range (Table 6). Serum triglyceride (TG), blood glucose (GLU), creatine (Cr), and total cholesterol (CHOL) in T1 rats were significantly lower than those in CK (*P* < *0*.05), but the difference with other treatment groups was not significant. Serum alkaline phosphatase (ALP) in T1 rats was significantly higher than those in CK, T2, and T3 (*P* < 0.05). Serum alanine aminotransferase (ALT) in T2 rats was significantly lower than that in CK (*P* < 0.05), and the difference with other treatment groups was not significant. Serum alanine aminotransferase (ALT) of T2 rats was significantly lower than that of CK (*P* < *0*.05), but the difference with other treatment groups was not significant. Total bilirubin (TBIL) in the serum of T2 rats was significantly lower than that of CK (*P* < 0.05), but the difference with other treatment groups was not significant.

**Table 6:** Effects of Moringa on blood biochemical indicators in rats during the experimental period.

Items	Treatments			
	CK	T1	T2	T3
ALP (U/l)	55.4 ± 7.54b	90.7 ± 9.47a	45.1 ± 7.31b	55.2 ± 10.33b
ALT (U/l)	50.74 ± 18.91a	42.71 ± 7.53ab	38.72 ± 4.87b	49.82 ± 8.2ab
AST (U/l)	161.15 ± 25.62a	158.78 ± 24.25a	126.51 ± 8.02ab	120.87 ± 13.82ab
TG (mmol/l)	0.89 ± 0.08a	0.59 ± 0.06bc	0.67 ± 0.06abc	0.64 ± 0.1abc
TBIL(µmol/L)	2.58 ± 0.32a	1.84 ± 0.32ab	1.76 ± 0.21b	2.17 ± 0.12ab
Cr (µmol/l)	46.34 ± 4.14a	33.15 ± 1.22b	39.4 ± 2.54ab	42.91 ± 6.93ab
BUN (µmol/l)	4.78 ± 0.23b	5.05 ± 0.37bc	5.38 ± 0.13bc	4.88 ± 0.17bc
GLU (mmol/l)	6.42 ± 0.46a	4.75 ± 0.19b	6.14 ± 0.32a	6.88 ± 0.34a
CHOL (mmol/l)	2.28 ± 0.21a	1.64 ± 0.19b	1.81 ± 0.22ab	2.15 ± 0.07ab
TP (g/l)	60.91 ± 2.18a	58.45 ± 2.77a	59.59 ± 1.91a	62.67 ± 1.12a
ALB (g/l)	34.46 ± 1.57a	32.87 ± 1.61a	34.6 ± 0.72a	34.38 ± 0.98a

The results were expressed as mean ± standard deviation and marked as significant level. Different lowercase letters in the same industry showed significant differences (*P* < 0.05).

*ALP* alkalinephosphatase, *TG* triglyceride, *ALT* alanine aminotransferase, *TBIL* total bilirubin, *Cr* creatinine, *BUN* blood urea nitrogen, *GLU* bloodglucose, *TP* total protein, *AST* aspartate amino transferase, *ALB* albumin, *CHOL* total cholesterol.

### Histopathology

**Figure 1:**
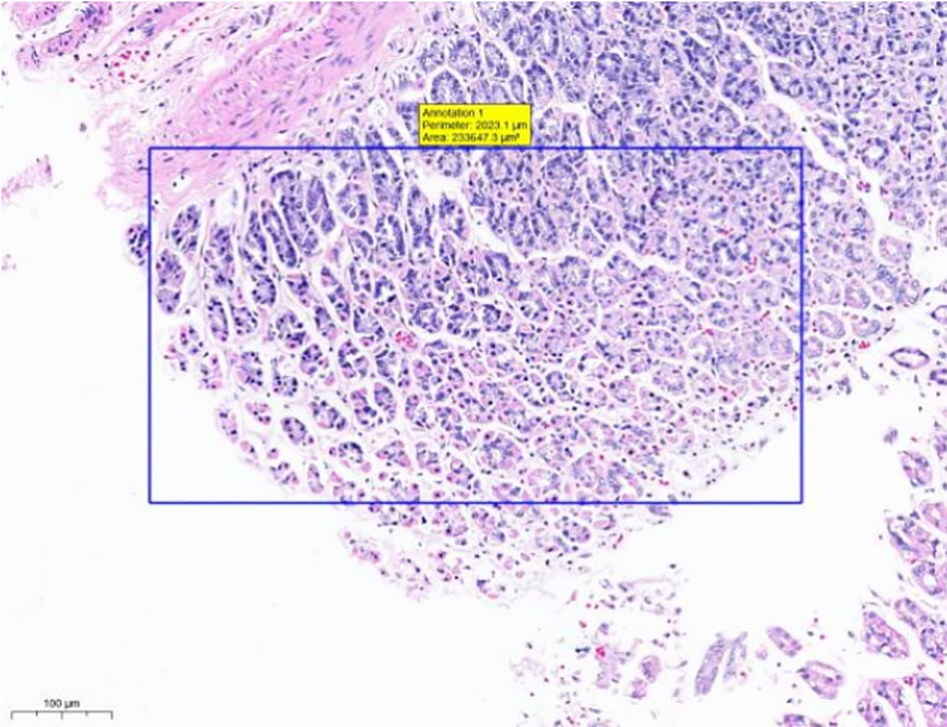
Stomach of CK group (H&E 13×).

**Figure 2:**
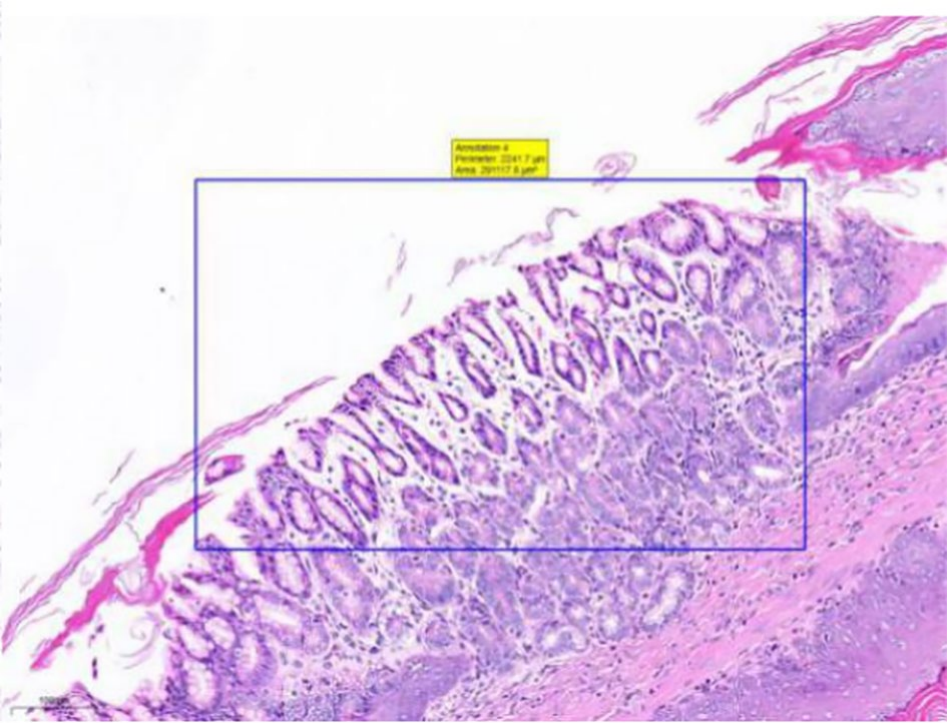
Stomach of T1 group (H&E 13×).

**Figure 3:**
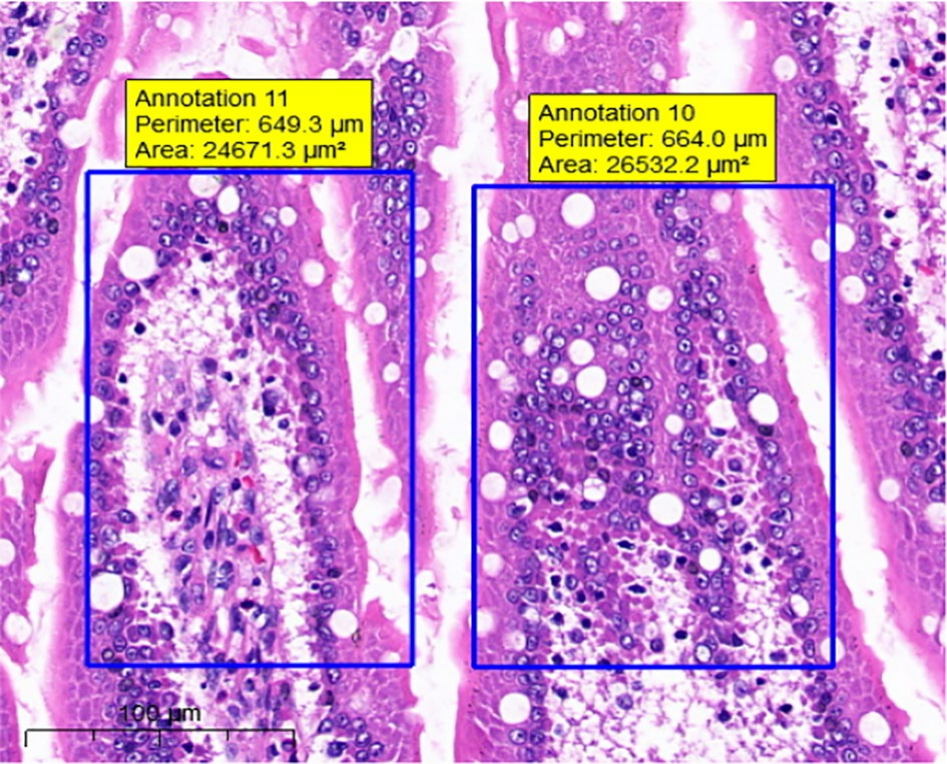
Group CK jejunum (H&E 15×).

**Figure 4:**
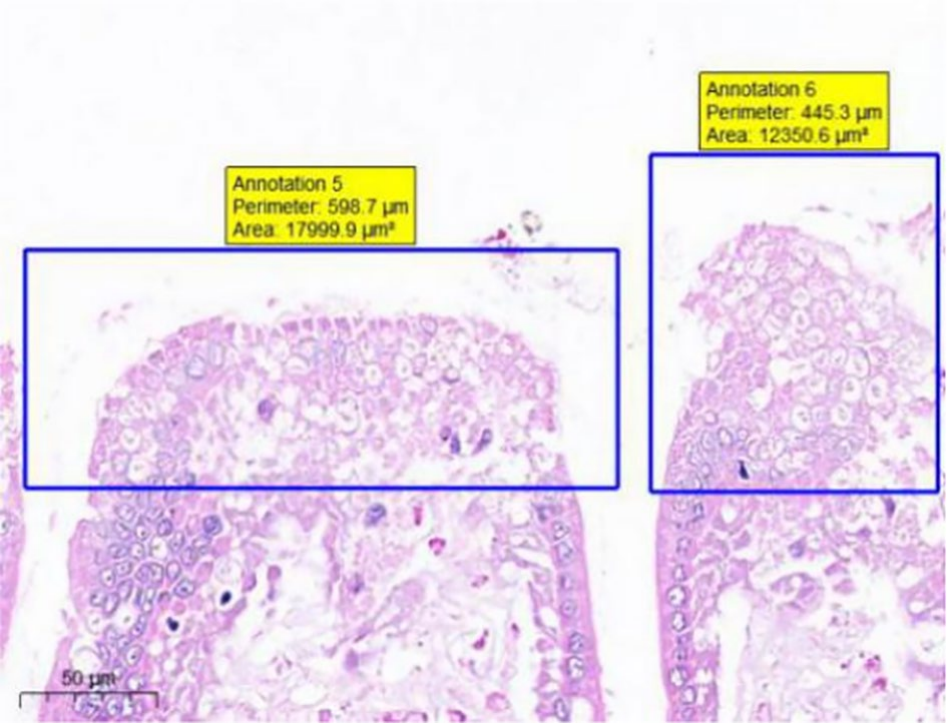
Group T1 jejunum (H&E 15×).

**Figure 5:**
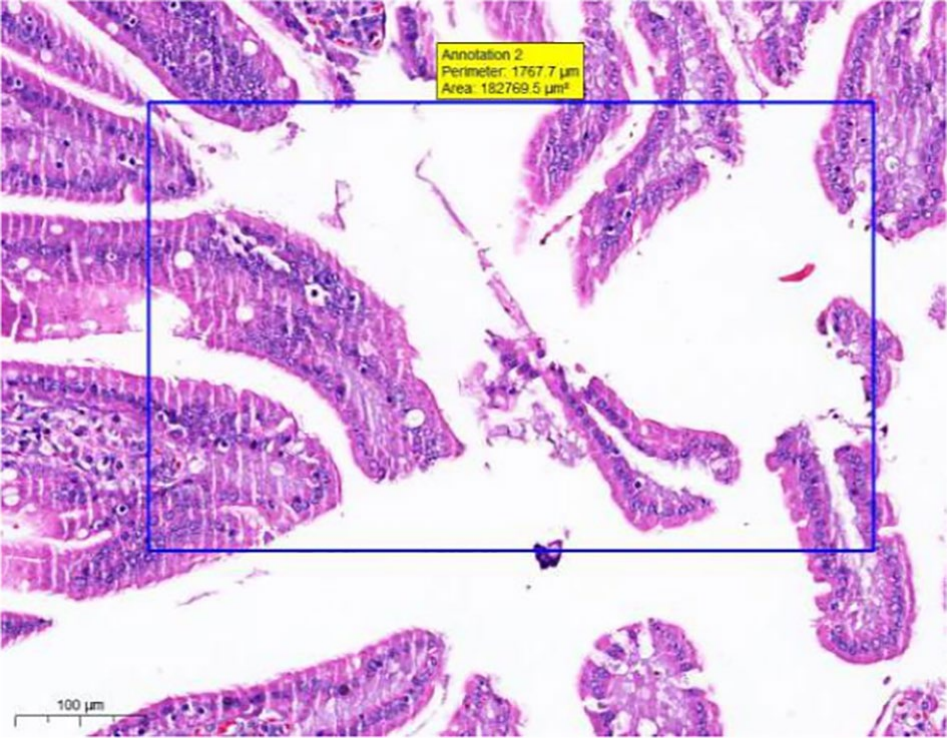
CK group jejunum (H&E 15×).

**Figure 6:**
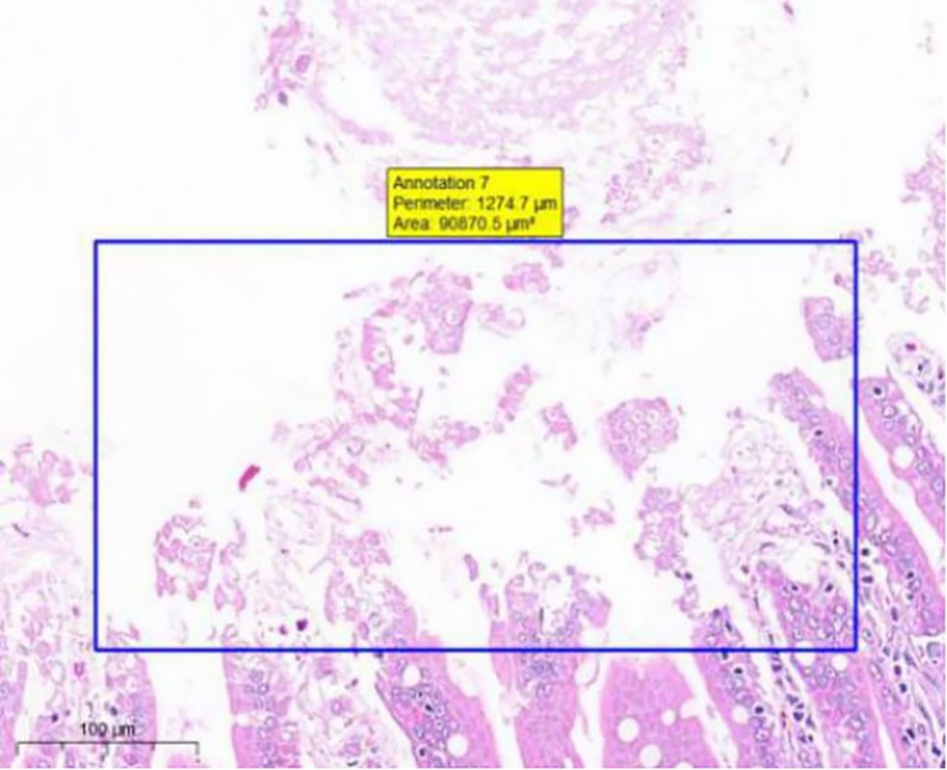
T1 group jejunum (H&E 15×).

**Figure 7:**
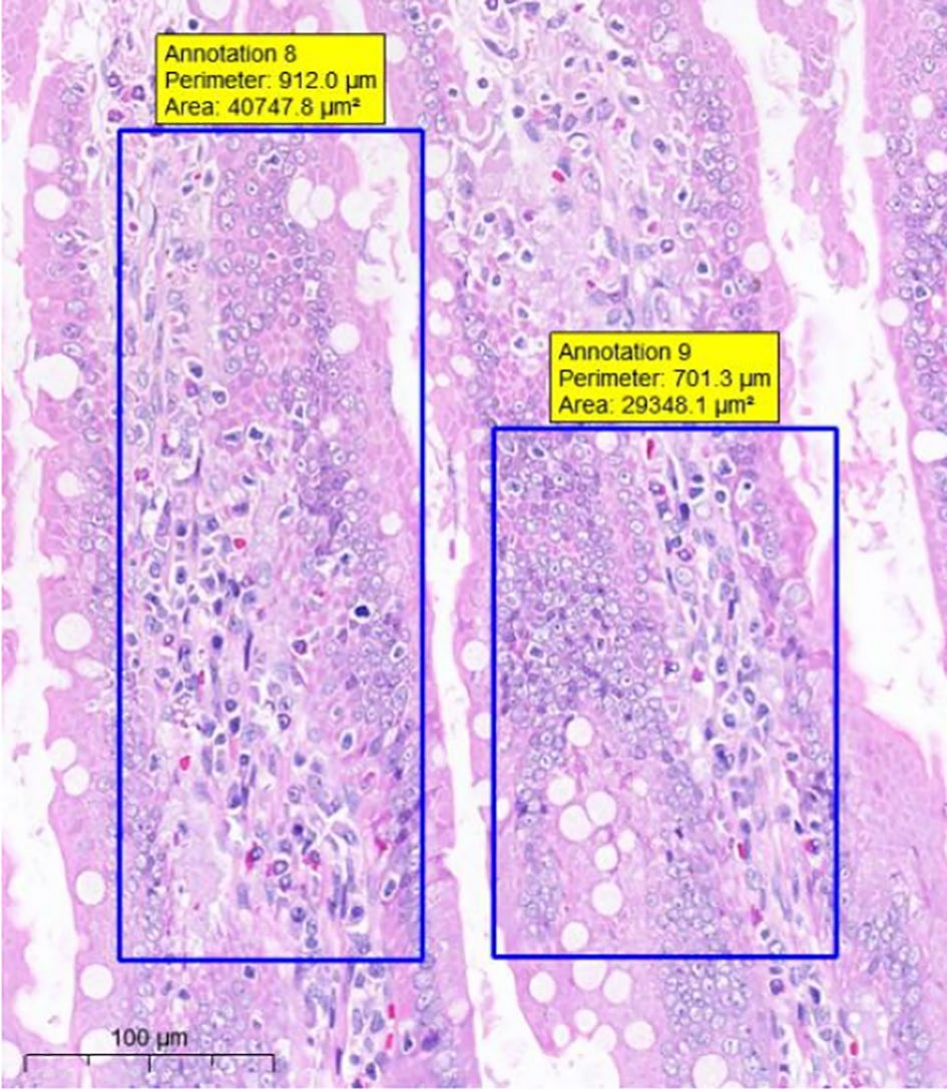
CK group jejunum (H&E 15×).

**Figure 8:**
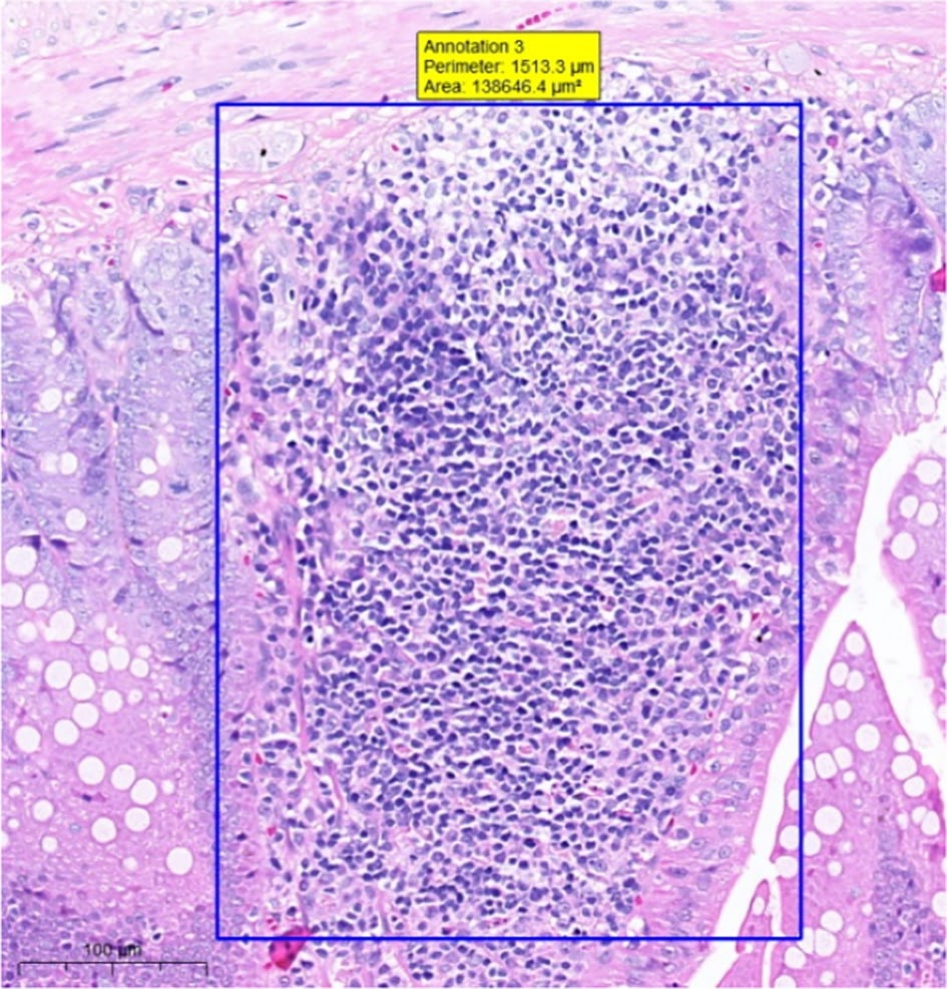
T1 group jejunum (H&E 15×).

At the end of the gavage period, the stomach of rats in the CK group (Fig. 1) and the gastric mucosal layer of rats in the T1 group were damaged. The surface cell nuclei were deeply stained, and the interstitial space between cells was significantly increased, forming partial active proliferation (Fig. 2). Compared with the jejunum of rats in the CK group (Fig. 3), the jejunum of rats in the T1 group showed no nuclear staining of superficial mucosal cells (Fig. 4), and a small number of superficial cells were shed compared with CK (Fig. 5), while the superficial cells were increased (Fig. 6) and enlarged compared to those in the jejunum of rats in the CK group (Fig. 7), forming a whole layer of cellular hyperplasia (Fig. 8). No abnormalities were seen in the ileum.

## Discussion

In this study, a 28-day subacute toxicity test was conducted on SD rats using low, medium, and high doses (1.5 g/kg, 3 g/kg, and 6 g/kg) of Moringa homogenate. The general behavioral condition, body weight, and food intake of rats in each group were recorded during the test period. Blood was collected at the end of the gavage period and analyzed for hematological and blood biochemical indices to evaluate the safety of Moringa. This experiment followed the “Chinese food 28-day oral toxicity test standard (GB15193.22-2014)”.

General behavioral status, body weight, and food intake are important indicators to characterize the dose-toxicity relationship of Moringa in rats^9–12^. Rats in the high-dose group of fresh Moringa homogenate showedrough coats, average mental status, normal movement and response, and diarrhea, while the medium and low-dose groups did not have diarrhea. This indicates that the oral administration of Moringa homogenate at 6 g/kg would have adverse effects on rats, while oral administration at 3 g/kg and below did not have any adverse effects on rats. The reason for diarrhea may be due to the high concentration in the high-dose group, which resulted in stretching of the stomach, behavioral adverse reactions, and mild diarrhea. It is also possible that the high dose of Moringa homogenate contained too high a concentration of a laxative component.

Body weight and ingestion status were more sensitive to the toxic effects of Moringa^13^. The body weight of rats in all groups tended to increase gradually with the duration of the gavage. Although the rats gavaged with high doses had diarrhea, the mean daily weight gain was not significantly different from that of the control group, demonstrating that high doses of fresh Moringa homogenate did not negatively affect the growth performance of rats. There was no significant difference in the food intake of rats gavaged with fresh Moringa homogenate in all treatment groups compared with the control group.

Blood plays an important role in maintaining normal metabolism and homeostasis in animals^14^. Any stimulus may affect the organism by causing changes in blood composition; therefore, blood tests are one of the most common and important tests in the diagnosis of animal diseases^15^. Animal hematology and blood biochemical indices are important and sensitive indicators of animal health and are essential in safety evaluation tests^16^. The hematological indices of the four groups of rats varied within the normal reference range. Platelet activity function is generally assessed by combining two parameters, PLT and MPV^17^. The blood PLT and MPV of rats in the fresh Moringa homogenate high-dose group were significantly lower than in the control group but still within the normal values and thus likely have no toxicological effect. High neutrophil levels are suggestive of inflammation, tissue damage, and pulmonary pathology^18^. The neutrophil levels in rats of all groups were within the normal range, indicating no toxicological effect. Red blood count (RBC), hemoglobin (HGB), and hematocrit (HCT) in the blood are important physiological indicators that respond to the characteristics of respiration, transport, and regulation of body fluid osmolality in animals^19^. No significant differences in RBC, HGB, and HCT were observed in any of the dose groups of rats compared with the control group, indicating that Moringa homogenate had no effect on the blood.

Serum biochemical parameters, including ALP, ALT, and aspartate aminotransferase (AST), are used to detect hepatobiliary diseases^20^. The dose of fresh Moringa homogenate in the T1 group caused significantly higher ALP in rats than in the control group. However, as AST, ALT, and TBIL in the T1 group were not significantly different from the control group, hepatobiliary diseases were excluded, and the values were presumed to be within the normal physiological range. TG, GLU, and CHOL were significantly lower in rats in the fresh Moringa homogenate high-dose group compared to the control group, indicating that fresh Moringa homogenate given to rats at 6 g/kg significantly reduced lipid, blood glucose, and cholesterol levels, which may be due to the high content of flavonoids in Moringa, which have a direct effect on blood glucose and cholesterol^21^. Cr and blood urea nitrogen (BUN) are important indicators of renal function. The administration of high doses of fresh Moringa significantly reduced Cr levels compared to controls, but BUN levels did not differ significantly from controls.

At the end of the gavage period, the thorax and abdomen of SD rats were grossly dissected and observed with the naked eye for size, color, and texture. No abnormal changes related to Moringa were observed. Histological observation of the heart, liver, spleen, lung, and kidney from rats in each treatment group showed no abnormalities, indicating no effect on the circulatory, hematological, immune, respiratory, and urinary systems of the rats. In contrast, the high dose (6 g/kg) of fresh Moringa homogenate had effects on the stomach and jejunum of rats compared to the control group. Some damage to the gastric mucosa layer was observed, probably due to the strong irritating odor and spicy taste of Moringa homogenate, which can cause damage to the gastric mucosa when consumed for a long time. It also led to deep staining of the nuclei of the superficial layer of cells in the stomach, the formation of some active proliferation, and a significant increase in the interstitial gap, which was caused by inflammation of the stomach in rats with diarrhea. No nuclear staining of superficial mucosal cells was observed in the jejunum, and a small amount of superficial cell detachment occurred as a result of indigestion or diarrhea.

## Conclusions

High doses (6 g/kg) of horseradish wood homogenate cause diarrhea in rats, while medium (3 g/kg) and low doses (1.5 g/kg) do not cause abnormal reactions in rats. No toxicological effects on the body weight, food intake, hematology, serum biochemical indicators, and main organs of rats were observed, but high doses of horseradish wood homogenate (6 g/kg) had an impact on the gastric mucosa tract of rats. Therefore, the safety threshold of Moringa homogenate should be ≤ 3 g/kg.

## Data Availability

Te data that support the fndings of this study are available from the frst author upon reasonable request. Experimental research and feld studies on plants, including the collection of plant material, comply with relevant institutional, national, and international guidelines and legislation.
